# Structural Characterizations and Biological Evaluation of a Natural Polysaccharide from Branches of *Camellia oleifera* Abel

**DOI:** 10.3390/ph18010051

**Published:** 2025-01-03

**Authors:** Shengjia Lu, Yali Zhang, Yanghui Ou, Jianghui Xin, Hongliang Yao, Litao Guan

**Affiliations:** 1 College of Materials and Energy, South China Agricultural University, Guangzhou 510642, China; 15123186435@163.com (S.L.); 18672222553@163.com (J.X.); 2 Guangdong Key Laboratory of Animal Conservation and Resource Utilization, Institute of Zoology, Guangdong Academy of Sciences, Guangzhou 510260, China; ouyh0807@gmail.com (Y.O.); yaohl@giz.gd.cn (H.Y.)

**Keywords:** anti-inflammation, food health, polysaccharide, *Camellia oleifera* Abel

## Abstract

Background: *Camellia oleifera* Abel (*C. oleifera*) is widely cultivated and serves as an important source of edible oil. Yet, during oil production, pruned branches generate significant waste and contribute to environmental pollution. Objectives: In this work, we obtain a natural polysaccharide from the branches of *C. oleifera* and optimize its extraction using Box–Behnken design (BBD), which is a statistical method commonly used in response surface methodology. Additionally, we study its properties, such as monosaccharide composition, structural features, antioxidant, and anti-inflammatory abilities. Results: BBD was employed to optimize polysaccharide extraction (solid-liquid ratio = 1:40; 90 °C; 130 min) for a higher yield. After purification, the major monosaccharides of branches of *C. oleifera’s* polysaccharide (CBP) were disclosed as glucose and galactose. Subsequent structural features of CBP were measured. The antioxidant and anti-inflammatory abilities were measured. The highly scavenging rates of the 2,2-diphenyl-1-picrylhydrazyl and hydroxyl radicals, with the chelating capacity of Fe^2+^, indicate potent antioxidant activity of CBP. Conclusions: In general, CBP demonstrated significant anti-inflammatory activity with down-regulating the expression of IL-6 and IL-1β in the LPS-induced macrophage RAW264.7 model. This bioactive polysaccharide adds value to waste branches by offering a novel approach to waste recycling and the development of *C. oleifera*.

## 1. Introduction

*Camellia oleifera* Abel (*C. oleifera*) is a small evergreen tree or shrub from the Camellia family, known for its medicinal and edible properties, and is primarily distributed in Asia [1]. The main function of this plant is to extract oil from its seeds. Due to its high oil yield, *C. oleifera* has become an important edible oil resource in China [2], driving its planting area to expand to 4.5 million hectares [3]. To enhance fruit production for oil extraction, large quantities of branches are pruned annually [4]. Yet, incineration or burying of disposed branches of *C. oleifera*, which is the normal way to handle this waste, can cause air pollution and wood waste. Therefore, recycling or extraction of certain bioactive components from those branches, such as polysaccharides of plants, can have better environmental affinities and benefits for medical utilization. 

Medical and edible polysaccharides possess numerous bioactivities and can be readily extracted from various plant sources, including crops, fruits, vegetables, and herbs, for applications in diverse medical fields. Natural plant polysaccharides commonly exhibit antioxidant, anti-tumor, antibacterial, hypoglycemic, and immunomodulatory activities [5,6,7]. Recent studies have demonstrated that polysaccharides have been isolated from various parts of *C. oleifera*, such as leaves, flowers, fruit shells, and seed cakes [8]. For instance, a polysaccharide derived from the fruit shell of *C. oleifera* has been shown to exert antioxidant activity by upregulating superoxide dismutase expression via DAF-16 activation [9]. The hypoglycemic effect in diabetic mice has been attributed to a polysaccharide extracted from the seed cake of *C. oleifera*, with its activity linked to the composition of monosaccharides [10].

However, the biological functions and molecular mechanisms of polysaccharides, especially those from the branches of *C. oleifera*—which are major components often discarded without use—remain poorly understood; and their study could uncover potential medical applications. In this study, we optimize the extraction methodologies of *C. oleifera* to increase yields of crude polysaccharide extracted from branches of *C. oleifera* (CCBP) and investigate the structural basis and biological mechanism of purified *Camellia oleifera* branch polysaccharide (CBP). This study identifies a natural polysaccharide extracted from the branches of *C. oleifera* that exhibits antioxidant and anti-inflammatory activities and holds potential for applications in health food development and waste wood recycling.

## 2. Results and Discussion

### 2.1. Extraction Process Optimization

#### 2.1.1. Single-Factor Experiment of CCBP

**Figure 1:**
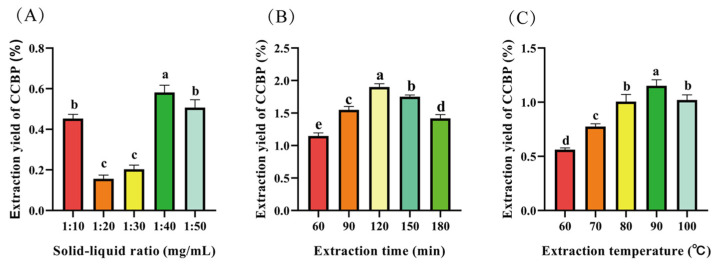
Single-factor experimental diagram of CCBP. (**A**) Effect of solid-liquid ratio, (**B**) extraction time, and (**C**) extraction temperature on extraction yield of CCBP. Data are mean ± SD (*n* = 3 replicates). Symbols above the columns (a, b, and c) indicated statistical significance (*p* < 0.05). The “a” represents the group with the highest extraction rate within this group and has significant differences from other groups. The “b” represents the group with the second highest extraction rate within this group. Similarly, the meanings of “c, d and e” are arranged in order. If there is no significant difference between the two groups, they are all classified as the same group. Comparison of a and b, *p* < 0.05; Comparison of b and c, *p* < 0.05; Comparison of b and c, *p* < 0.05, and so on.

The extraction of CCBP (crude *Camellia oleifera* branch polysaccharide) was optimized by evaluating the solid-liquid ratio, extraction time, and extraction temperature through single-factor analysis (Figure 1A–C).

At solid-liquid ratios of 1:10 or 1:50 g/mL, the extraction yield of CCBP was significantly higher than at ratios of 1:20 and 1:30 g/mL (*p* < 0.05, Figure 1A). The highest CCBP yield was observed at a solid-liquid ratio of 1:40, which was significantly higher than at 1:10 and 1:50, indicating that the transfer driving force of polysaccharides reached its maximum [11].

As the temperature increased from 60 to 100 °C (Figure 1B), the extraction yield rose, peaking at 90 °C, before gradually decreasing. The extraction temperature for *C. oleifera* is typically set at 80 °C [12], but in this study, 90 °C was used to significantly enhance the extraction yield. This phenomenon may be attributed to the gradual increase in plant polysaccharides with rising extraction temperatures [4]. However, when the extraction temperature is too high, the high temperature will break the polymerization degree of polysaccharides, leading to their decomposition and thus reducing the extraction rate.

Another influential factor in the extraction field of CCBP is extraction time. When the extraction time increased from 60 to 180 min (Figure 1C) at 90 °C and a solid-liquid ratio of 1:40, the maximum extraction yield reached 1.9%. After peaking at 120 min, the yield began to decrease slightly. Excessive extraction time of polysaccharides at higher temperatures can lead to polysaccharide hydrolysis, which may be the reason for the decrease in polysaccharide yield after 120 min [13]. The optimal time range for extraction is set between 90 min and 150 min, which can save the phenomenon of excessive extraction time, energy waste, and low yield.

#### 2.1.2. Response Surface Methodology

As described in Section 2.1.1, the solid-liquid ratio, extraction temperature, and extraction time are the key variables that significantly influence the yield of CCBP. Consequently, to design and optimize the process variables for maximizing the yield of CCBP, this study utilized the three-factor, three-level Box–Behnken Design (BBD) within the Response Surface Methodology (RSM). The experimental data and technology variables were displayed in the Supplementary Material (Table S1). The yield of CCBP ranged from 0.51% to 1.95%, with the maximum yield of 1.95% achieved at a solid-liquid ratio of 1:40 (g/mL), 90 °C, and 120 min. The following second-order polynomial step-by-step equation was obtained using multivariate regression analysis of the experimental data and was represented by equation:

Y = 1.89 + 0.0305A + 0.1439B + 0.1806C − 0.0485AB + 0.0175AC − 0.2562BC − 0.9121A^2^ − 0.2859B^2^ − 0.2094C^2^

ANOVA was used to analyze the significance and serviceability of the model. A summary of statistical data are presented in Table 1. The coefficient of determination (R^2^) for the fitting model is 0.9962, indicating that only 0.38% of the total variation is not explained by the model. The adjusted coefficient of determination (Adj-R^2^) is 0.9914, further confirming the high significance of the model. The monomial coefficients (B and C), quadratic term coefficients (A^2^, B^2^, and C^2^), and the interaction term coefficient (BC) are significant, while the other parameter coefficients are not. The validity of the model is proved because the F-value for the lack of fit is insignificant (*p* > 0.05). The model demonstrates high precision, reliability, and reproducibility, as indicated by the low coefficient of variation (C.V.) of 2.51 [14]. Therefore, the interaction between extraction temperature (B) and extraction temperature (C) significantly affects the process of CCBP extraction, which should be given full consideration.

**Figure 2:**
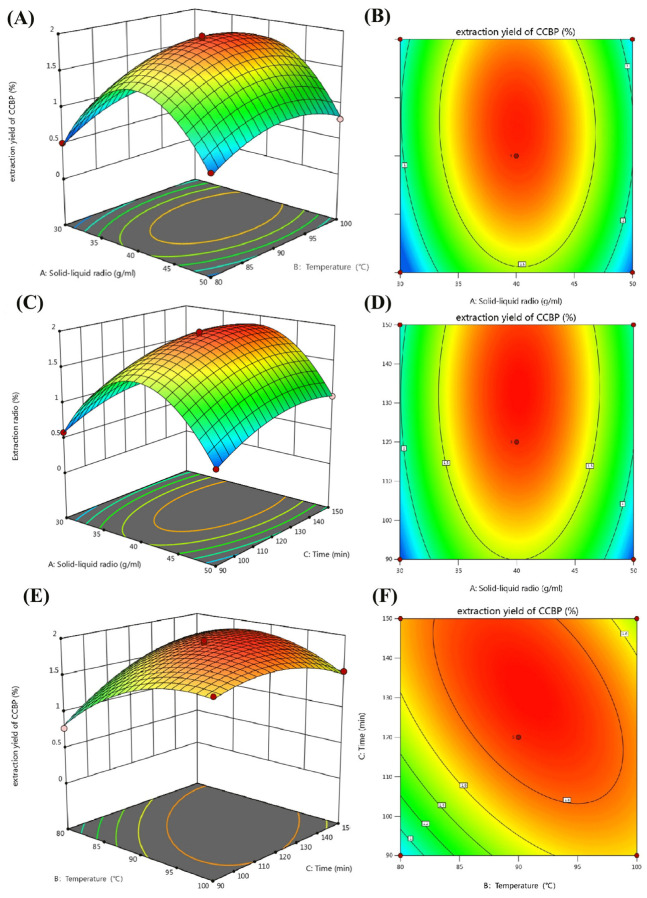
Response surface diagram of CCBP. (**A**,**C**,**E**) Response surface of 3D images and contour plots (**B**,**D**,**F**) showing the effect of ratio of solid-liquid ratio ((**A**), g/mL), extraction temperature ((**B**), °C), and extraction time ((**C**), min) on the extraction yield of CCBP (Y1, %).

The 3D response surface and 2D contour plots presented in Figure 2A–F illustrate the relationship and interaction between the process variables. An increase in two variables (B and C) caused an initial increase in the Y1 of CCBP (Figure 2E,F), and then a decrease was observed. The maximum Y1 of CCBP was achieved at an extraction temperature of 90 °C and an extraction time of 120 min. Table 1 and Figure 2 show that the interaction between BC was stronger than between other interaction variables (AB and AC).

According to the analysis of variance (Table 1) and the response surface diagram (Figure 2A–F), the numerical optimization results show that under this system, the best optimized conditions are as follows: Solid-liquid ratio of 1:40.187 (g/mL), extraction temperature of 90.77 °C, and extraction time of 131.553 min. Under the best optimized conditions, the predicted yield of CCBP predicted is 1.935%. Considering the realistic experimental conditions, this experiment uses conditions close to the predicted standard (Solid-liquid ratio of 1:40, 90 °C, and 130 min). The final extraction rate of CCBP is 1.901 ± 0.02%. Experiment Y1 is close to the predicted value, indicating the validity of the BBD model.

### 2.2. Purification of CBP and Chemical Composition Analysis

**Figure 3:**
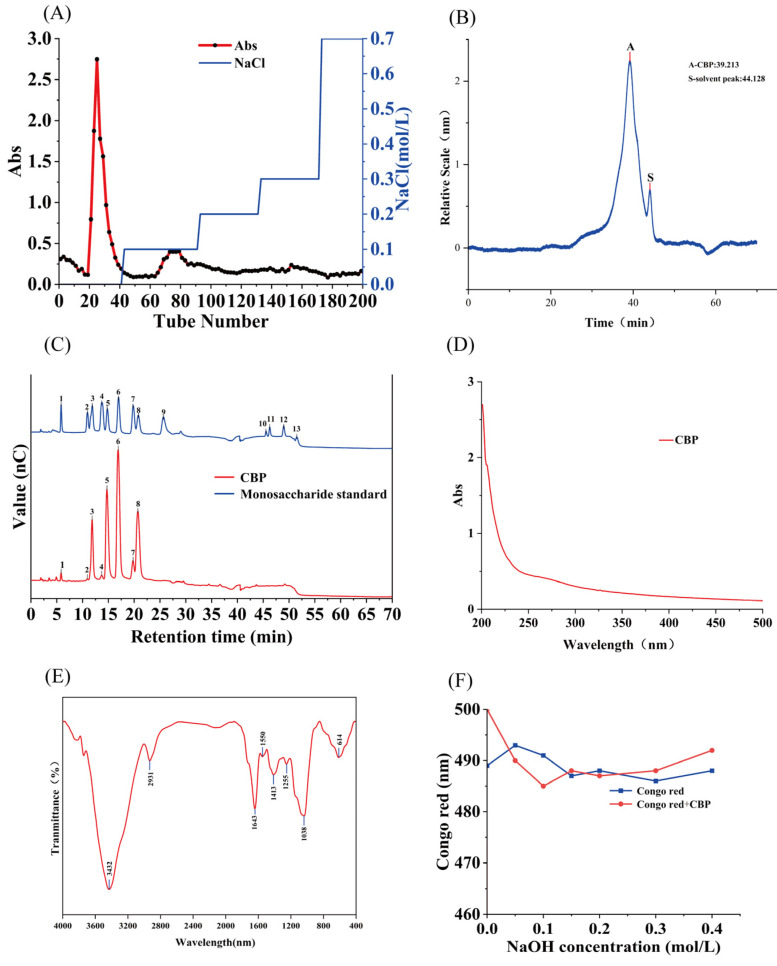
Chemical property of CBP. (**A**) Elution curve of CCBP on Experdex-75 column; (**B**) HPGPC spectrum and peak Mw of CBP; (**C**) HPLC chromatograms of standard monosaccharides and CBP; 1, Fucose; 2, Galactosamine Hydrochloride; 3, Arabinose; 4, Glucosamine Hydrochloride; 5, Galactose; 6, Glucose; 7, Xylose; 8, Mannose; 9, Ribose; 10, Galacturonic acid; 11, Guluronic acid; 12, Glucuronic acid; 13, Mannuronic acid; (**D**) UV spectra of CBP; (**E**) FT-IR of CBP; (**F**) Congo red experimental analysis of CBP.

CCBP was purified by experdex-75 ion exchange chromatography and eluted with distilled water to collect CBP (Figure 3A). The contents of total sugar, protein, and uronic acid in CBP are 75.77 ± 2.9%, 1.14 ± 0.53%, and 26.75 ± 0.55%. The results suggest that the polysaccharides were further purified and that CBP may be an acidic polysaccharide.

### 2.3. Determination of Molecular Weight and Monosaccharide Composition Analysis

The main Mw of CBP is 10,548 Da. The monosaccharide composition of CBP is shown in Figure 3C. Results show that CBP is composed of fucose, galactosamine hydrochloride, arabinose, glucosamine hydrochloride, galactose, glucose, xylose, and mannose in a molar ratio of 0.018:0.002:0.127:0.006:0.273:0.277:0.043:0.253.

### 2.4. Structure Characteristics of CBP

#### 2.4.1. UV Spectroscopy and IR Spectroscopy

The absence of absorption peaks in the UV-VIS spectrum at 260 nm and 280 nm confirms the absence of proteins or nucleic acids in CBP (Figure 3D) [15]. Figure 3E shows the FT-IR of CBP. 3432 cm^−1^ shows a deep and broad absorption peak due to the inter- and intra-molecular hydrogen bonds that cause strength vibration of -OH. The stretching vibration of OH groups usually exhibits an absorption peak at 3600–3200 nm^−1^ [16]. The -CH stretching vibration causes a weak peak at around 2931 nm^−1^. The two bands 3432 cm^−1^ and 2931 cm^−1^ are related to the characteristic absorbance of the polysaccharide [17]. The absorbance at 1643 cm^−1^ is due to the symmetric stretching of free carboxyl groups [12]. The peak at around 1550 cm^−1^ indicates a possible stretching vibration of C-N [18]. In addition, bands at about 1250 cm^−1^ correspond to asymmetrical stretching of C-O-C [19]. The Ssugar characteristic absorption peak, which is also a typical IR signal of glucan, causes the peak near 1038 cm^−1^. Due to the asymmetric stretching vibration of the ether bond on the sugar ring, the phenomenon appeared [20]. The absorbance at 614 nm^−1^ is related to the “skeletal region”. The “skeletal region” is the region blowing 800 nm^−1^ Bands in this region have a bearing on the carbohydrate skeletal vibrations [21].

#### 2.4.2. Scanning Electron Microscopy of CBP

**Figure 4:**
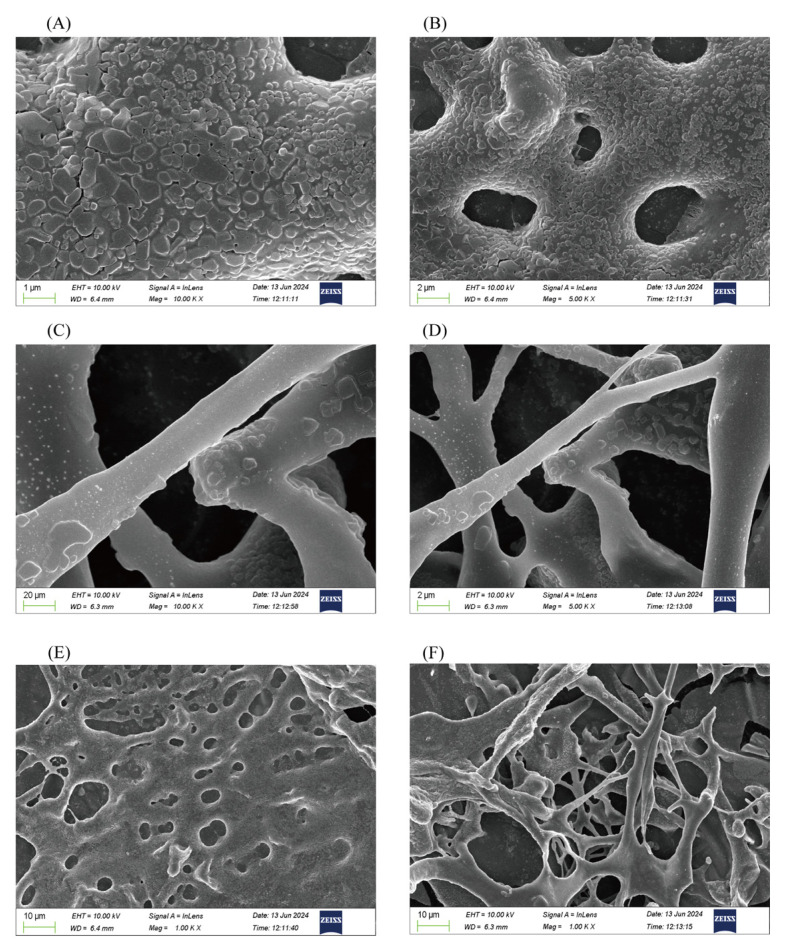
Surface morphology features of CBP. Scanning electron microscopy (**A**–**F**) of CBP; In alphabetical order, magnifications are (10.0 kX, 5.0 kX, 10.0 kX, 5.0 kX, 1.0 kX, 1.0 kX).

The surface morphology of CBP is shown in Figure 4A–F. CBP exhibits an irregularly layered structure with a relatively rough surface and large pore size. The overall spatial structure is relatively loose, resembling a “tree branch” shape. These results suggest that CBP exhibits strong intermolecular interactions.

#### 2.4.3. Atomic Force Microscopy of CBP

**Figure 5:**
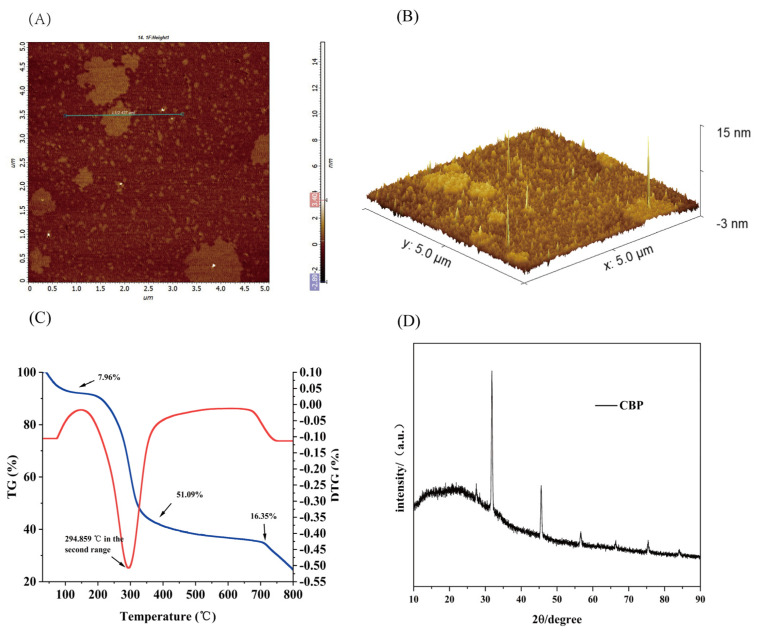
Morphological features and physical properties of CBP. (**A**,**B**) Atomic Force Microscopy; (**C**) The thermal stability of CBP. Blue line: TG; Red line: DTG; (**D**) X-Ray Diffraction.

The morphology images (planar Figure 5A and 3D stereogram Figure 5B) show the CBP sample exhibits multiple branched side-connected structures. Furthermore, the “mountain-like” shape observed in the 3D image suggests that the polysaccharide macromolecules have a highly branched chemical structure and relatively rough surface features [22].

#### 2.4.4. X-Ray Diffraction of CBP

One commonly used method for examining the structure of polysaccharides is X-ray diffraction (XRD). Its primary purpose is to test and analyze the crystalline structure of materials. The X-ray diffraction pattern can reveal the tensile strength, flexibility, swelling behavior, solubility, and other physical properties of polysaccharides [23]. The X-ray diffraction pattern of the extracted CBP is shown in Figure 5D. In the region of 2θ = 32.25° and 41.56°, two peaks can be observed. The crystalline index of the extracted polysaccharide was 23.64%, which was used by Origin 2024 software to analyze the graph of XRD. By studying the data collected from the graph, the main polysaccharides extracted from branches of *C. oleifera* are proved to have low overall crystallinity, indicating they have a semi-crystalline structure [24].

#### 2.4.5. Congo Red Assay of the CBP

The maximum absorption of CBP and Congo Red adding increasing concentration NaOH solution is shown in Figure 3F. The maximum absorption wavelengths of CBP show no significant red shift, indicating that CBP lacks a triple-helix structure [25].

### 2.5. Thermal Analysis of the CBP

The thermal stability of polysaccharides is typically evaluated using thermogravimetry (TG) and derivative thermogravimetry (DTG) methods [26]. TG and DTG testing curves show the three weight loss stages of CBP (Figure 5C). In the initial weight loss stage of CBP (30 °C to 250 °C), the weight loss rate is 7.96%, indicating the evaporation of water in CBP [27]. In the second weight loss stage (250 °C to 450 °C), the weight loss rate is 51.09%, which may be the result of the depolymerization of polysaccharide structure [28]. In the third stage of weight loss (450 °C to 800 °C), CBP exhibits a weight loss rate of 16.35%. The DTG curve indicates that CBP exhibits a maximum weight loss rate of 0.51% at 294.85 °C. A decrease in polysaccharide Mw corresponds to a reduction in thermal stability.

### 2.6. Determination of Antioxidant and Anti-Inflammatory Abilities

#### 2.6.1. 2,2-Diphenyl-1-Picrylhydrazyl (DPPH) Radical Scavenging of CBP

**Figure 6:**
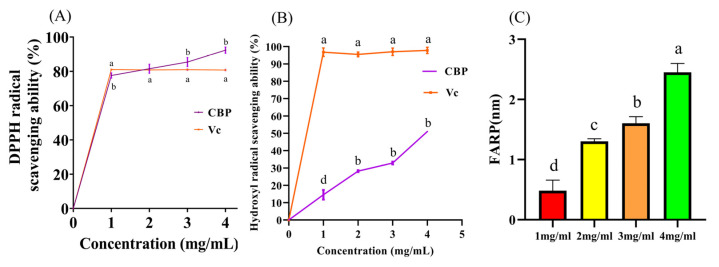
Antioxidant capacity of CBP. (**A**) DPPH radical scavenging activity of CBP; (**B**) Hydroxyl radical scavenging activity of CBP; (**C**) Ferric reducing antioxidant power of CBP. Different letters (a, b, c, and d) at the different concentrations indicate a statistically significant difference (*p* < 0.05). In (**A**,**B**), “a” and “b” represent the significant differences between Vc and CBP at the same concentration. ‘a’ represents the Vc curve, ‘b’ represents the CBP curve; In (**C**), the “a” represents the group with the highest oxidation ability in this group, and has significant differences compared to other groups. The “b” represents the group with the second strongest oxidation ability in the group. Similarly, the meanings of “c and d” are arranged in order. If there is no significant difference between the two groups, they are classified as the same group. Comparison of a and b, *p* < 0.05; Comparison of b and c, *p* < 0.05; Comparison of b and c, *p* < 0.05, and so on.

The occurrence of degenerative processes is closely linked to high levels of free radicals in molecular biology. Cellular dysfunction, aging, and diseases are proven to relate to the oxidative stress that is caused by high levels of free radicals [19,29]. Therefore, a vital index to evaluate the polysaccharide’s biological activity is antioxidant capacity. One simple, fast, and reliable method for detecting the antioxidant properties of natural products is the DPPH scavenging assay [30]. The scavenging activity of DPPH free radicals increases with the CBP concentration (0–4 mg/mL), as shown in Figure 6A. At a total of 4 mg/mL, CBP could effectively remove 92.35% ± 1.74. As the concentration increases, almost the same antioxidant effect of VC can be achieved.

#### 2.6.2. Hydroxyl Radical Scavenging of CBP

Almost any biomolecule in living cells has free radical chain reaction according to the hydroxyl radical, which is the most harmful free radical for an organism [31]. CBP can scavenge the hydroxyl radical, which is shown in Figure 6B. This ability is correlated with concentration, and as the concentration increases, the clearance ability also increases until it reaches 4 mg/mL. The best scavenging effect on the hydroxyl radical reaches 51.04% ± 0.37. The hydroxyl radical scavenging activity of CBP could be related to the lower molecular weight and monosaccharide composition [32].

#### 2.6.3. Ferric-Reducing Antioxidant Power (FARP) of CBP

Redox-active transition metals, such as iron (Fe), are essential for the survival and growth of mammals. However, various oxidative damages are related to the free radical formation catalyzed by these metals [33]. One of the antioxidant efficacy predictors is determining the reducing power [34]. The main mechanism is that Fe^3+^ is reduced to Fe^2+^ after interacting with drugs of antioxidant activity [35]. Increasing the concentration of CBP continuously enhances its chelating effects, as shown in Figure 6C. At 4 mg/mL, the chelating effect of CBP reaches 2.45 mM ± 0.14. The relatively high uronic acid content and low protein impurities in CBP may enhance its chelating ability [36].

#### 2.6.4. In Vitro Anti-Inflammatory Activity of CBP

**Figure 7:**
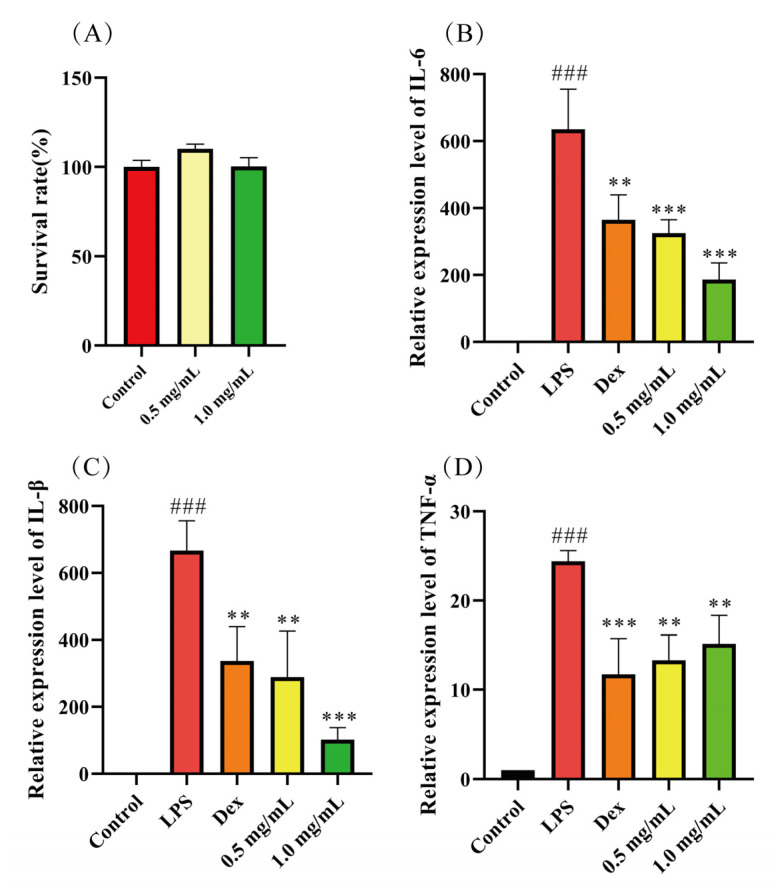
Anti-inflammatory ability of CBP. (**A**) Effect of CBP on cell viability of RAW264.7 macrophages; Effect of CBP on the relative expression of IL-6 (**B**), IL-β (**C**), and TNF-α (**D**) in RAW264.7 macrophages induced by LPS. Compared with Blank group, ### *p* < 0.001; compared with LPS group, ** *p* < 0.025, *** *p* < 0.001. The above values are expressed as mean ± SD (*n* = 3).

Cytokines play a critical role as mediators in various physiological and pathological processes, including inflammation and immune responses [37]. Upon stimulation by LPS (Lipopolysaccharides), macrophages produce various inflammatory mediators, including IL-6, IL-1β, and TNF-α [38]. Therefore, in this study, the anti-inflammatory activity of CBP on RAW264.7 macrophages was evaluated by detecting the expression of IL-6, as shown in Figure 7A,B. Both DEX (Dexamethasone) and CBP significantly reduced IL-6 expression compared to the LPS group (*p* < 0.05 or *p*< 0.001) (Figure 7A). Compared to the LPS group, both DEX and CBP significantly reduced IL-6 expression (*p* < 0.05 or 0.001). The effect became more pronounced with increasing concentrations of CBP. Both DEX and CBP significantly inhibited IL-1β expression compared to the LPS group (*p* < 0.05 or 0.001), with the effect becoming more pronounced at higher concentrations. In the TNF-α expression study, both treatments exhibited some inhibitory effects compared to the LPS group; however, the effects were not particularly significant (*p* < 0.05) and lacked a clear concentration-dependent relationship. This may be related to the higher total sugar content of CBP [39] and its monosaccharide composition [40]. Lu et al. extracted Rehmannia qlutinosa polysaccharides with significant anti-inflammatory activity, identifying Gal and Glcas as their primary components [41]. These monosaccharides are similar to those primarily found in CBP, which predominantly contains Gal and is associated with its anti-inflammatory effects [42]. Additionally, a higher proportion of Man has also been linked to the anti-inflammatory effects of polysaccharides [43]. Therefore, CBP demonstrates significant anti-inflammatory activity and holds promising potential for development into anti-inflammatory drugs from waste material.

## 3. Materials and Methods

### 3.1. Biological Materials and Chemicals

The branches of *C. oleifera* were collected from Changtang, Shaoguan City, in spring. They were dried at 55 °C using a drying box (DHG-9240, Shanghai Yiheng Technology Co., Ltd., Shanghai, China), subsequently crushed into powder using a blender (JYL-C020E, Jiuyang Co., Ltd., Jinan, Shandong, China), and stored at 25 °C. Kits for hydroxyl radicals (#S0116), DPPH radicals (#2621), and total antioxidant capacity (#1720) were all purchased from Enzyme Linked Biotechnology Co., Ltd. (Shanghai, China).

The cell culture medium (the RAW264.7 mouse macrophage cell) was obtained from Wuhan Pricella Biotechnology Co., Ltd. (Wuhan, China), and qPCR reagents (#Q711-02) were obtained from Nanjing Vazyme Biotechnology Co., Ltd. (Nanjing, China). Lipopolysaccharide, LPS (#L2880), and dexamethasone, Dex (#D1756), obtained from Merck Co., Ltd. (Darmstadt, Germany). All other chemicals and solvents were of laboratory grade and were used directly.

### 3.2. Acquisition and Purification of Polysaccharides

The CCBP was prepared using a traditional hot water extraction method [12]. Briefly, impurities of CCBP were removed, which included pigments, lipids, and oligo saccharides, after the raw materials were extracted with a hot-water method based on Box–Behnken design. The original volume of sample supernatant was concentrated by 75%, and anhydrous ethanol was added overnight at 4 °C to derive precipitation of water extraction. After that, the protein of the precipitation was removed, dialyzed, and freeze-dried to derive crude polysaccharides.

CCBP dissolved in ionized water was filtered in a 0.45 μM membrane, and the filtered solution was added into ion exchange chromatography columns to collect chromatography solution. The content of carbohydrates in the chromatography solution was determined using the phenol-sulfuric acid method. After the purified solution of polysaccharide was collected, dialyzed, and freeze-dried, CBP was derived.

### 3.3. The Steps of Extraction Process Optimization

#### 3.3.1. Single Factor Experiment

Previous extraction steps in Method 2.2 were comprised of three factors and a five-level simplex lattice design, which included solid-liquid ratios (1:10, 1:20, 1:30, 1:40, 1:50 g/mL), temperature (60, 70, 80, 90, 100 °C), and time (60, 90, 120, 150, 180 min). The extraction yield of CCBP (freeze-dried powder state) was used to determine the optimized experimental conditions.

#### 3.3.2. Response Surface Methodology Optimization Process

According to the optimized experimental condition, these assays were comprised of a three-parameter, three-level design, which included solid-liquid ratio (g/mL, A), temperature (°C, B), and time (min, C). Data of specified ranges used Design-Expert software version 13.0 (Stat-Ease Inc., Minneapolis, MN, USA) to collect 17 group experimental results, which were presented in Table S1.

### 3.4. Chemical Composition Content Analysis

The phenol-sulfuric acid method [44], Bradford method [45], and sulfate-carbazole method [46] were used to test the content of the total sugars, proteins, and uronic acid of the CBP. The Standard samples use dextrose, bovine serum albumin (BSA), and D-galacturonic acid. The extraction yield of CBP (EY, %) was presented as:

$$EY\;(\%)=\frac{M1}{M2}\;\times 100\%$$

where M1 is the mass of the lyophilized CBP and M2 is the quality of the raw powder.

### 3.5. Determination of Molecular Weight

CBP (5 mg) was dissolved in NaCl solution (0.05 mol/L). The solution was measured by High-performance gel permeation chromatography (HPGPC) using a 20 A difference detector (Shimadzu, Kyoto, Japan) and a BRT105-103-101 tandem gel column (8 × 300 mm) [47].

### 3.6. Monosaccharide Composition Analysis

CBP (5 mg) mixes TFA (2 ml, 2 mol/L). Then, CBP was hydrolyzed for 2 h at 120 °C. The sample solution was dried using nitrogen gas. High-performance ion exchange chromatography (HPIC) of the CBP was measured using a Dionex Carbopac™ PA20 column (3 × 150 mm, Thermo Fisher Scientific, Waltham, MA, USA) and an electrochemistry detector (ICS5000, Thermo Fisher Scientific, Waltham, MA, USA) [48].

### 3.7. Ultraviolet-Visible Spectroscopy and Infrared Spectroscopy

An aqueous solution of CBP was prepared, and the UV spectrum of CBP was analyzed in the 200 to 500 nm range using an ultraviolet-visible (UV-VIS) spectrophotometer (NP80 Touch, Implen, Munich, Germany) and was used to determine the presence of protein residues. The lyophilized CBP samples were homogeneously mixed with ground KBr pellets and were tested by Fourier transform infrared spectrophotometers (Bruker Corporation, Saarbrucken, Germany) in the frequency range of 400–4000 cm^−1^.

### 3.8. Scanning Electron Microscopy Assay

A scanning electron microscope (Sigma-300, Carl Zeiss AG, Oberkochen, Germany) was used to observe the surface morphology of the CBP.

### 3.9. Atomic Force Microscopy Assay

A sample of CBP (5 mg) was stirred, dissolved in ionized water, and placed in a 50 mL volumetric flask. A solution of CBP (5 mL) was diluted to 50 mL using ionized water. A drop of sample solution was placed on the peeled mica sheet and was dried at room temperature. The sample was tested using an atomic force microscope. (Dimension Edge, Bruker Corporation, Saarbrucken, Germany)

### 3.10. X-Ray Diffraction Assay

Appropriated samples of CBP were placed on the X-ray diffraction detection plate and fixed with ethanol on the surface. Another sample of CBP was taken as a control. The crystal structure was measured using an X-ray powder diffractometer. (ULTIMAIV, Rigaku, Tokyo, Japan) Scanning conditions: The range of 2θ is 5° to 90°, the scanning speed is 2°/min, and I = 20 mA [49]. The crystallinity percentage was calculated according to the following equation:

$$\mathrm{Crystallinity}\;\mathrm{Index}\;=\;(\frac{Crystalline\;area}{Crystalline\;area\;+\;amorphous\;area})\;\times \;100\%$$

### 3.11. Congo Red Assay of the CBP

The solution of CBP (0.25 mg/mL) was mixed with 80 µM Congo Red. The mixed solution was sequentially added NaOH solution (0, 0.05, 0.1, 0.15, 0.2, 0.3, 0.4 mol/mL). The maximum absorption of wavelength was measured using UV-VIS (NP80 Touch, Implement, Munich, Germany spectrophotometer) in the scope of 400–600 nm.

### 3.12. Thermal Analysis of the CBP

An analyzer of thermal (Mettler TGA/DSC^3+^, Zurich, Switzerland) manufactured by Mettler Toledo based on TG-DTG was used to study the thermal properties of the CBP. Briefly, the CBP (10 mg) was added to Al_2_O_3_, and aluminum of empty weight was used as a basic material. Temperature gradually increased from 30 °C to 800 °C in a nitrogen environment at a heating rate of 10 °C/min [50].

### 3.13. In Vitro Antioxidant Activity

#### 3.13.1. DPPH Radical Scavenging Assay

A solution of CBP (1–4 mg/mL, 10 mL) was mixed with a working solution (0.10 mL). After the mixed solution was placed in the dark at room temperature for 30 min, absorbance was measured at 515 nm using a microplate reader (Tecan M2001, Tecan, Mannedorf, Switzerland) [43]. Vitamin C (Vc) was used as a positive control. The calculation formula to determine DPPH radical scavenging rate is as follows:

$$DPPH\mathrm{radical}\;\mathrm{scavenging}\;\mathrm{rate}\;(\%)=\frac{A1-A2}{A1}\;\times \;100\%$$

where A1 represents the absorbance measurements of blank controls in the working solution, and A2 represents absorbance measurements of the samples.

#### 3.13.2. Hydroxyl Radical Scavenging Assay

A solution of CBP (1–4 mg/mL) was mixed with a working solution and buffer. Absorbance was measured at 536 nm using a microplate reader after incubation for 60 min at 37 °C. Vc was used as a positive control. The calculation formula to determine the hydroxyl radical scavenging rate is as follows:

(1) $$\mathrm{Hydroxyl}\;\mathrm{radical}\;\mathrm{scavening}\;\mathrm{rate}\;(\%)=\frac{Ak-Ai}{Aj-Ai}\;\times \;100\%$$

In the formula: Ak represents the absorbance measurements of the sample; Aj represents the absorbance measurements of the care tube; and Ai represents the absorbance measurements of the blank tube.

#### 3.13.3. Ferric-Reducing Antioxidant Power

According to the method of the Total Antioxidant Capacity Detection Kit [51], a solution of CBP (1–4 mg/mL, 5 µL) was mixed with 180 µL of FRAP working solution, and then the mixed solution was incubated at 25 °C for 5 min to measure the absorbance at 593 nm. The antioxidant capacity of CBP is calculated based on the FeSO_4_ standard curve.

### 3.14. Cell Viability Assay

RAW 264.7 macrophages were seeded on 96-well plates, and 10 μL of CBP at a range of concentrations was added as control groups for 24 h. Cell culture-medium cell counting kit-8 (CCK-8) was added to treated cells, and absorbance was measured at 490 nm wavelength using a microplate reader (Tecan M2001, Thermo Fisher, Waltham, MA, USA) to evaluate cell viability (the test has three parallel experiments).

### 3.15. Inflammatory Factor Assay

RAW264.7 macrophages were seeded to 12-well culture plates. The study included four groups: (1) control (no LPS stimulation), (2) LPS stimulation (1 µg/mL LPS), (3) treatment (0.5–1 mg/mL CBP + 1 µg/mL LPS), (4) positive controls (1 µM DEX + 1 µg/mL LPS). Cells were harvested for subsequent extraction of RNA assays.

### 3.16. Quantitative PCR Assay

RNA was extracted using the RNA extraction kit (#RC112-01), and cDNA was transcribed using real-time fluorescence quantitative PCR according to Vazyme’s method after induction with 1 µg/mL LPS for 4 h [52].

The primers were synthesized by Sangon Biotech Co., Ltd. (Shanghai, China), and the sequences are presented in the  Supplementary Material (Table S2).

### 3.17. Experimental Data Statistics and Analysis

All data are indicated as mean ± SD and are collected from a minimum of three separate experiments. Sizes of sample and numbers of control for each experiment are determined using statistical analysis and are described for each single experimental component. The statistical analysis was carried out using the *t*-test and one-way analysis of variance (ANOVA). *p* < 0.05 was considered as statistical significance.

## 4. Conclusions

This study obtained CCBP through optimized hot water extraction from *C. oleifera* branches and purified it using a cellulose column to isolate the polysaccharide CBP. CBP exhibited strong antioxidant and anti-inflammatory activities. This study provides a new environmentally friendly approach for utilizing discarded *C. oleifera* branches and enhancing their added value. Thus, recycling polysaccharides from discarded wood branches could contribute to food health applications in the near future.

## Figures and Tables

**Table 1:** ANOVA for the response surface quadratic model of CCBP yield.

Source	CCBP					
	Sum of Squares	df	Mean Square	F-Value	*p*-Value	
Model	5.00	9	0.5559	204.82	<0.0001	significant
A-Solid-liquid ratio	0.0074	1	0.0074	2.74	0.1417	
B-Temperature	0.1656	1	0.1656	61.01	0.0001	
C-Time	0.2610	1	0.2610	96.16	<0.0001	
AB	0.0094	1	0.0094	3.47	0.1049	
AC	0.0012	1	0.0012	0.4513	0.5233	
BC	0.2627	1	0.2627	96.77	<0.0001	
A^2^	3.50	1	3.50	1290.58	<0.0001	
B^2^	0.3441	1	0.3441	126.77	<0.0001	
C^2^	0.1846	1	0.1846	68.00	<0.0001	
Residual	0.0190	7	0.0027			
Lack of Fit	0.0114	3	0.0038	2.01	0.2545	not significant
Pure Error	0.0076	4	0.0019	Pure Error	0.0076	
Cor. Total	5.02	16		Cor Total	5.02	
R^2^ = 0.9962, R^2^ adj = 0.9914, R^2^ pre = 0.9612, C.V.% = 4.23						

## Data Availability

Data are contained within the article and the Supplementary Material.

## Supplementary Materials

The following supporting information can be downloaded at: www.mdpi.com/article/10.3390/ph18010051/s1, Figure S1: Comparison of Fresh Branches and Processed Dried Branches Before and After Treatment; Figure S2: The H-NMR spectrum of CBP; Table S1: All group experimental results of Response Surface Methodology; Table S2: Primers used in this study; Table S3: Actual polysaccharide extraction of yield under optimized conditions. Tables S1–S3 were used in the study.
