# Supplementary material for: Structural Characterizations and Biological Evaluation of a Natural Polysaccharide from Branches of Camellia oleifera Abel
Source: Pharmaceuticals (Basel). 2025 Jan 3;18(1):51. doi: 10.3390/ph18010051 (PMC11769495; doi:10.3390/ph18010051)

**Table S1** All group experimental results of Response Surface Methodology

| Entry | Coded variable levels | | | Yield (%) |
|---|---|---|---|---|
| | $A$ [a] | $B$ [a] | $C$ [a] | |
| 1 | 1:30 | 80 | 120 | 0.51 |
| 2 | 1:50 | 80 | 120 | 0.669 |
| 3 | 1：30 | 100 | 120 | 0.82 |
| 4 | 1：50 | 100 | 120 | 0.785 |
| 5 | 1：30 | 90 | 90 | 0.585 |
| 6 | 1：50 | 90 | 90 | 0.61 |
| 7 | 1：30 | 90 | 150 | 0.9 |
| 8 | 1：50 | 90 | 150 | 0.995 |
| 9 | 1：40 | 80 | 90 | 0.775 |
| 10 | 1：40 | 100 | 90 | 1.65 |
| 11 | 1: 40 | 80 | 150 | 1.66 |
| 12 | 1: 40 | 100 | 150 | 1.51 |
| 13 | 1: 40 | 90 | 120 | 1.925 |
| 14 | 1: 40 | 90 | 120 | 1.88 |
| 15 | 1: 40 | 90 | 120 | 1.95 |
| 16 | 1: 40 | 90 | 120 | 1.84 |
| 17 | 1: 40 | 90 | 120 | 1.875 |

$A^a$, $B^a$ and $C^a$ denote the solid-to-liquid ratio (g/mL), extraction temperature (℃) and extraction time (min), respectively.

**Table S2** Primers used in this study.

| Target gene | Forward sequence (5'→3') | Reverse sequence (5'→3') |
|---|---|---|
| IL-1β | TCGTGCTGTCGGACCCATAT | GTCGTTGCTTGGTTCTCCTTGT |
| IL-6 | TACTCGGCAAACCTAGTGCG | GTGTCCCAACATTCATATTGTCAGT |
| TNF-α | GGGGATTATGGCTCAGGGTC | CGAGGCTCCAGTGAATTCGG |
| β-Actin | GGCTGTATTCCCCTCCATCG | CCAGTTGGTAACAATGCCATGT |

**Table S3** Actual polysaccharide extraction of yield under optimized conditions

| Entry | Actual usage standards | | | Yield (%) | standard deviation |
|---|---|---|---|---|---|
| | $A$ [a] | $B$ [a] | $C$ [a] | | |
| 1 | 1: 40 | 90 | 130 | 1.925 | |
| 2 | 1: 40 | 90 | 130 | 1.875 | 0.025166115 |
| 3 | 1: 40 | 90 | 130 | 1.905 | |

$A^a$, $B^a$ and $C^a$ denote the solid-to-liquid ratio (g/mL), extraction temperature (℃) and extraction time (min), respectively.

**Figure S1**

**Comparison of Fresh Branches and Processed Dried Branches Before and After Treatment**

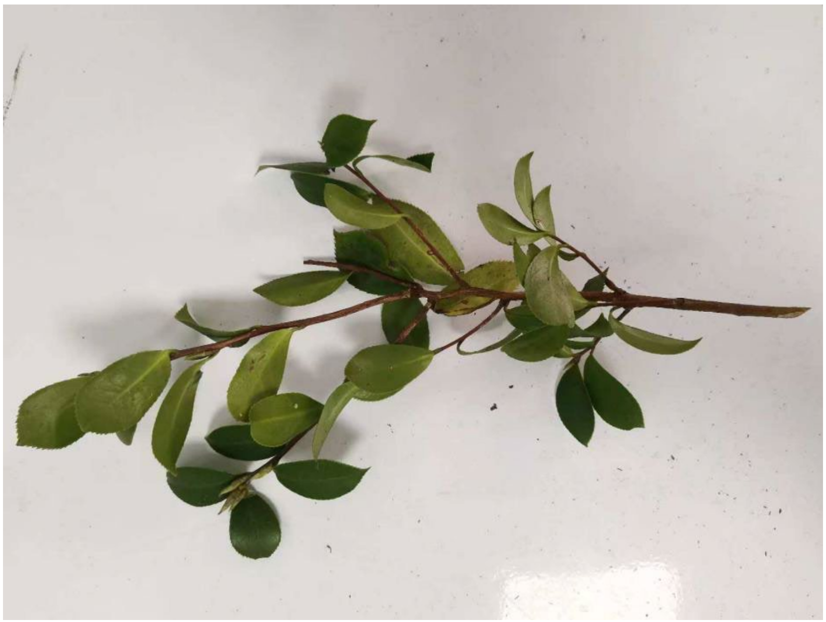

**Fresh branch of Camellia oleifera Abel**

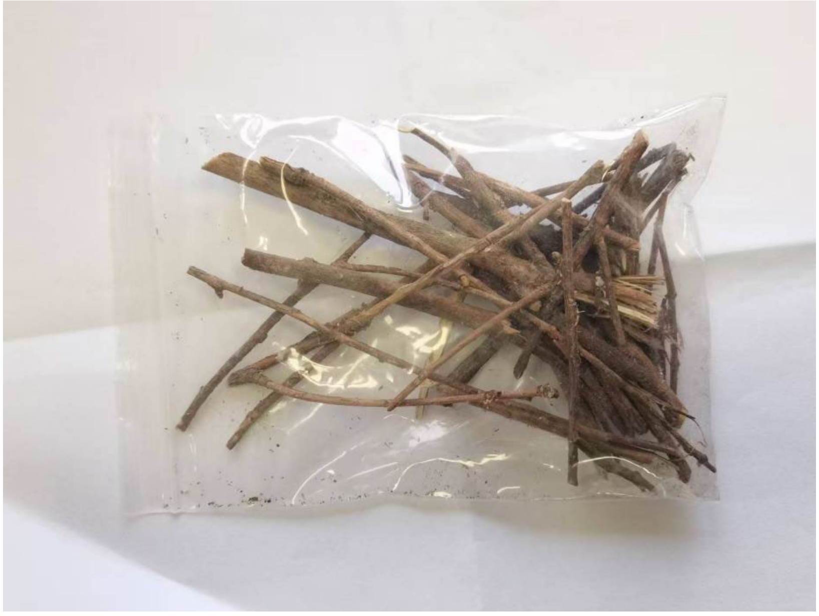

**Leaves removed and dried branches**

# Figure S2 The H-NMR spectrum of CBP

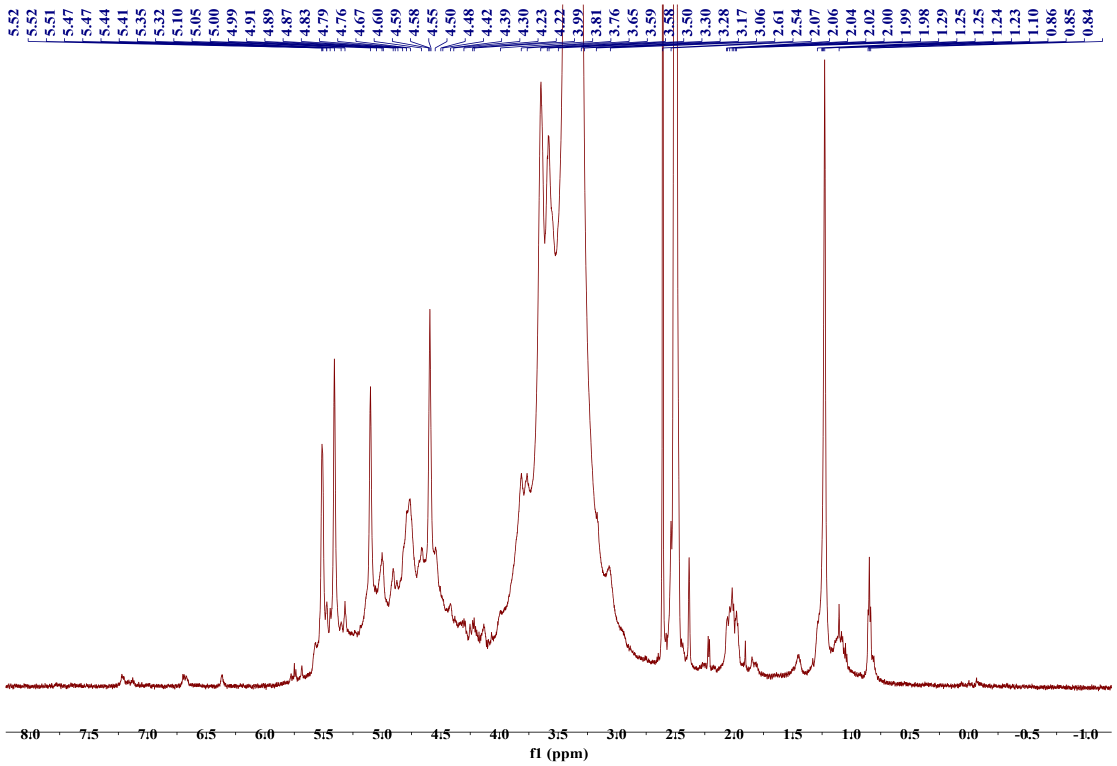

Supplement: Supplementary file 1 [file pharmaceuticals-18-00051-s001.zip › pharmaceuticals-3345640-supplementary.pdf]
